# Long-Acting Injectable Drugs in the Maintenance Therapy of Patients with Schizophrenia

**DOI:** 10.17650/2712-7672-2020-1-2-53-62

**Published:** 2020-12-04

**Authors:** Natalia N. Petrova, Valeria S. Serazetdinova

**Affiliations:** Saint Petersburg State University

**Keywords:** personal recovery, schizophrenia, long-acting injectable, paliperidone palmitate 1-month formulation, paliperidone palmitate 3-month formulation, remission, relapse, tolerability, личностное восстановление, шизофрения, инъекционные пролонги, палиперидон пальмитат 1 раз в месяц, палиперидон пальмитат 1 раз в 3 месяца

## Abstract

This article discusses case reports of treatment with paliperidone palmitate in comparison with data from recent publications. Second-generation long-acting injectable antipsychotics have been shown to provide better control of psychiatric manifestations, reduce the severity of negative symptoms, improve social functioning and quality of life of patients and relatives, and reduce the burden of disease for both the healthcare system and the caregivers. The case reports presented in this article demonstrate better quality of remission in schizophrenia patients treated with one- monthly and three-monthly paliperidone palmitate formulations, due to higher effi in preventing relapses, better safety and good tolerability regardless of patient age.

## INTRODUCTION

Recovery and satisfactory quality of life are the criteria for the effectiveness of modern therapy for schizophrenia. The ultimate treatment goals are to improve functional capacity, minimize residual symptoms and reduce the frequency and duration of relapses, since each relapse potentially worsens prognosis. Continuity of maintenance treatment is essential for a long-term positive outcome in patients with schizophrenia. Antipsychotics are the basis of schizophrenia treatment and long-acting injectable antipsychotics are designed to increase the effectiveness of therapy for schizophrenia spectrum disorders.

One representative of the atypical long-acting antipsychotics group is paliperidone palmitate, available in injectable suspension dosage form with a dosing schedule of once per month (PP1M). In the Russian Federation, this form of the medication is produced under the brand name Xeplion. The paliperidone molecule is the active metabolite of risperidone. By avoiding hepatic metabolism, paliperidone has acquired distinctive properties that provide a better tolerability and safety profile while retaining higher efficacy. A modern delivery system of paliperidone palmitate enables maintenance of the plasma concentration of paliperidone within the therapeutic range for up to five weeks and eliminates fluctuations in the drug levels. If the dose is missed, the level of the drug decreases slowly, creating a safety net in the case of patient non-compliance which thus gives time for medical intervention and minimizes potential adverse events.

Data from a multicentre study of PP1M (n = 645) indicated significant reductions in the frequency and duration of hospitalizations of patients with schizophrenia and schizophrenia spectrum disorders [1]. These data showed that Xeplion therapy is associated with better treatment adherence of outpatients [2], which is essential for long-term continuous therapy of schizophrenia.

A new second-generation long-acting injectable antipsychotic agent emerged in 2016. Trevicta, a three- monthly injectable formulation of paliperidone palmitate (PP3M), is the only product that offers an extended three- month 'window' of stable plasma levels. It is an ester of paliperidone and palmitic acid for intramuscular administration once every three months. It contains the same active substance as PP1M and is made by the same technology, but has a different strength and particle size, enabling the three-monthly administration schedule. PP3M is indicated for patients who are clinically stable and who have been adequately treated with PP1M for at least four months before switching to PP3M [3]. It is suggested that PP3M offers a substantially longer dosing interval compared with other long-acting formulations [4].

The efficacy of PP3M (175-525 mg) was similar both in Europeans and non-Europeans when it was compared with PP1M (50-150 mg) in 487 European adult (18-70 years) patients (PP3M, n = 242; PPIM, n = 245) and 508 non-European adult patients (PP3M, n = 241; PPIM, n = 267) with schizophrenia. However, the rate of adverse events was lower in Europeans (56% with PP3M, 59% with PP1M) than in non-Europeans (80% with PP3M, 73% with PP1M). The most common increase in body weight was in non-Europeans, especially in the Asian population [5].

The safety and efficacy of PP3M have been demonstrated in double-blind randomized clinical trials [6,7]. However, there is a need to better understand treatment regimens with PP3M in real world settings. From May 2014 until September 2016, a retrospective cohort study was conducted examining the treatment of adult patients with schizophrenia via documentation in the Symphony Health Solutions (SHS) database. The study included two cohorts: all patients with PP3M prescriptions (first cohort) and patients transferred from PP1M to PP3M according to a strict clinical transfer protocol (second cohort). Every four months and over a 12-month period, PP1M treatment regimens, the proportion of days covered by psychotropic drugs and health resource use patterns were evaluated. It was found that out of 1545 patients with PP3M (first cohort), 68.8% transferred from PP1M to PP3M (forming the second cohort). In both cohorts, the proportion of patients with the number of days covered by psychotropic medications (≥80% for antipsychotics, antidepressants, anxiolytics and mood stabilizing agents) increased. The proportion of patients making one or more visits to the emergency department, inpatient or outpatient departments decreased with the start of PP3M therapy. Among the patients followed up for four months or more after the first dose, 85-88% received the second dose; among the patients followed up for four months or more after the second dose, 87-90% received the third dose. PP3M was usually prescribed to patients with schizophrenia in accordance with the instructions. The most common first PP3M dose was 819 mg (the highest available) and most patients received a constant dose with the first three injections. The average number of days between subsequent PP3M statements was also consistent with the recommendations. The main reason for not transitioning as recommended to PP3M was the presence of gaps greater than 45 days in PP1M four months prior to PP3M initiation. A high proportion of the patients were in stable condition and were adherent to the treatment with PP3M [8].

PP3M has been approved for use in the long-term maintenance treatment of patients with schizophrenia who are clinically stable on PP1M therapy for at least four months. Since the available data confirm the efficacy and tolerability of PP3M compared to PP1M and placebo, PP3M should be considered as an appropriate treatment option for the patients who received maintenance therapy with PP1M [9]. It should be noted that these recommendations are followed in real clinical practice. Moreover, most patients who are prescribed paliperidone palmitate have already had exposure to risperidone, including its long-acting form. Consecutive transfer to PP1M and PP3M is accompanied by improvement of the clinical and functional characteristics of remission.

The practice of transfer from two-week long-acting injectables to PP1M and PP3M is in line with the current clinical guidelines. Garcfa-Carmona et al. (2020) compared PP1M and PP3M with two-week long-acting injectables for the following outcomes: the number of rehospitalizations, the number of the verified suicidal actions/attempts and the use of concomitant treatment, including benzodiazepines, oral antipsychotics and biperiden. A total of 431 patients diagnosed with schizophrenia spectrum disorder who had received the appropriate treatment over at least 12 months were evaluated. The results showed a significant decrease in the number of rehospitalizations during PP3M therapy compared to two-week injectables and aripiprazole once a month, while no significant differences in suicidal behaviour were found. There was a significantly lower level of benzodiazepine consumption in the PP1M and PP3M groups compared to the two-week injectables group. In addition, patients who received PP1M and PP3M medications used a significantly lower dose of haloperidol equivalent compared to the patients who received two-week long-acting injectables. Finally, significantly higher doses of biperiden were used by the two-week long-acting injectables group. These data indicate the advantages of PP1M and PP3M over two- week long-acting injectables [10].

A comparative analysis was conducted of the treatment results of PP1M and PP3M in patients with schizophrenia (n = >1100) who were taking and not taking oral risperidone/paliperidone (RIS/PALI). Results indicated that there was an improvement in mental state as assessed by the Positive and Negative Syndrome Scale (PANSS), both during and after relapse. The tolerability of the therapy was comparable regardless of the prior treatment of RIS/PALI [11].

It should be emphasized that PP3M has a number of advantages in addition to better adherence [12]. Symptomatic remission and a decrease in the severity of both positive and negative symptoms reflect the stability of treatment with PP3M. Moreover, significant functional remission, reduced frequency of drug administration and freedom from daily monitoring of the patient have a positive effect on the patient and reduce the burdensome aspects of their care. PP3M is a valuable antipsychotic treatment option that deserves consideration in terms of its broader role in the longterm treatment of schizophrenia; its utility should not be limited to prescribing in patients with poor adherence or when oral antipsychotics have failed.

Compared to two-week long-acting injectable antipsychotics, patients treated with PP1M and PP3M had a reduction in the number of rehospitalizations and concomitant medications [10]. This is consistent with information from domestic clinical practice showing improvements in extrapyramidal symptoms following transition to paliperidone palmitate therapy and the absence of the need for correctors.

Pharmacokinetic and pharmacodynamic models were designed to investigate the relationship between plasma concentrations of paliperidone and the risk of schizophrenia relapse in patients treated with PP1M and PP3M. In study A, patients (n = 305) were randomized to PP3M or placebo in the double-blind phase; in study B, patients (n = 1002) were randomized to PP3M or PP1M in the double-blind phase. Risk of relapse decreased with higher paliperidone concentrations for both PP1M and PP3M. Risk of relapse increased in patients with a higher number of previous hospitalizations and/or with a higher pre-randomization paliperidone concentration (study A) and in patients using concomitant benzodiazepine medication. The analysis confirmed that both PP1M and PP3M are comparable in preventing relapse [13].

Negative symptoms in schizophrenia are associated with impairments in social and cognitive functioning, leading to substantial long-term disability. A study investigating the impact of PP1M and PP3M on negative symptoms randomized patients (n = 1,016) to receive PP3M (n = 504) or PP1M (n = 512). Treatment with PP3M or PP1M demonstrated comparable improvement regarding negative symptoms in patients with moderate to severe negative symptoms and patients with prominent negative symptoms. Long-term treatment with PP3M demonstrated benefit, suggesting that continuous antipsychotic medication treatment for over one year is needed to achieve greater benefits for negative symptoms [14].

We now present several cases of the use of second- generation long-acting injectable antipsychotics to illustrate our own experience of PP1M and PP3M use.

### CLINICAL CASE 1*

Patient R., born in 1981 (age 39) in Leningrad. She has no known family history of mental disorders, however, according to the patient's mother, the father has pronounced characterological features, he is aggressive and displays violent behaviour. The patient also has an elder brother who is healthy. The patient's parents have been divorced since she was 16 years old. The patient did not have any disturbances of early development or adolescence. Menstruation started from the age of 14, with a regular cycle. The patient did not have any difficulties in social adjustment when she was a child. She attended preschool institutions, then school from the age of seven, she graduated from nine classes with an average academic performance and her favourite subject was French. After school, she worked as a checkout operator, but since 2008 she has been unemployed, with a lifetime disability group classification. She is single and does not have children.

The patient first noted changes in her mental state at the age of 16, when she became withdrawn and unsociable after her parents divorced. She refused food, hid it under her pillow, lost too much weight and did not explain her behaviour in any way. At the insistence of her mother, she was examined by a private psychiatrist and was treated for two months on an outpatient basis, taking medications. Her mental state improved and she started to eat. According to the patient, at that time a psychotic state was diagnosed. However, she decided not to take maintenance therapy. At that time, her attitude towards her mother changed dramatically, she reported that 'I began to hate her', 'I used obscenities towards her' and 'I wanted her to die'. She blamed mother for her father leaving the family, because 'I loved him very much, despite his violent behaviour'. The condition worsened again at the age of 17, with presentation of similar symptoms. The patient again refused to eat, became withdrawn-'blah'-and called her mother 'every name in the book'. She was examined by a private psychiatrist and took thioridazine for two months with further recommendation that she be monitored by a district psychiatrist. However, she did not go to a psychoneurological dispensary (outpatient community mental health clinic), as according to the patient, 'her condition has improved'. During this period, her father returned to the family and she communicated only with him, ignoring her mother. At the age of 18, she got a job as a store assistant. During this period, she became gentler in her relationship with her mother. After working for four years, she quit, explaining that 'there are a lot of non-Russians, they can cheat and not give money?. She started to think that she was being watched and followed everywhere. She got a job in another store, but only worked for a few months. Subsequently, she changed places of work frequently. Since the age of 25 (since 2006), she has not worked regularly anywhere, explaining that it is too heatty a load. According to the patient, she 'began to hate everyone', was suspicious, and refused to fill in CV forms when looking for a new job, because she believed that 'these data could be used against her'. She lost interest in previous activities, lost acquaintances and withdrew into herself. A year later, her aggressive behaviour towards her mother returned in verbal form, as she obscenely scolded her mother. From the beginning of spring 2008, she claimed that 'the air in the house is bad' because her mother's 'deodorant is especially poisonous' and believed that her mother put 'something' in her food. Since May 2008, she stopped leaving the house, it seemed to her that she was being Watched from a vacant lot', that there was video surveillance in the apartment; she turned off the phone and drew the curtains. She was involuntarily admitted to a psychiatric hospital for two and a half months (May to the end of July). Upon admission, she expressed delusional ideas of exposure and poisoning. She strained her ears as if she were hearing something and looked like she suffered from hallucinations, asking doctors 'do you feel this smell, as if they want to poison us?' and 'are you in cahoots with them?'. She shouted, was agitated, tense, angry and generally negatively predisposed. She stated that 'there are cameras everywhere at home'. She did not express any recognition of her condition. She was taking zuclopentixol at 20 mg per day. Her condition improved and quickly stabilized, with a reduction in the hallucinatory-delusional symptoms. In the ward she kept to herself, did not communicate with other patients and did not make plans for the future. Diagnosis according to ICD-10: F20.0 Paranoid schizophrenia.

Immediately following discharge from hospital, the patient's condition remained stable, but she felt sleepy and apathetic. She failed to find a job. Over time, she began to suffer from muscle stiffness. When trying to reduce the dosage of zuclopentixol to 10 mg per day, she again became irritable, aggressive towards her mother and expressed delusional ideas of poisoning. She was referred to a psychoneurological dispensary, where she was treated from October 2008 to February 2009. Diagnosis: F20.0 Paranoid schizophrenia. Disease course type: shift-like protracted delusional syndrome. Moderate apathia and abulia syndrome.

In the psychoneurological dispensary the patient received therapy via atypical antipsychotics (amisulpride, clozapine and risperidone). Her condition improved, her behaviour became more organized, the delusional symptoms resolved and the extrapyramidal symptoms reduced. She was then referred to rehabilitation department, where she attended occupation therapy groups and performed uncomplicated work, but coped very slowly. She was referred for medical and social expert evaluation and assigned to disability/ group II for one year.** She was discharged from the rehabilitation department with a recommendation to continue taking medication (risperidone 4 mg per day, trihexyphenidyl 4 mg per day). After a few months, she began to take the recommended medications on an irregular basis. In March 2009, she was transferred to therapy with long-acting haloperidol (intramuscular solution) with injections of 50 mg once every 14 days. On this therapy she noted an increase in apathy, indifference and lack of initiative. During this period, she got a low-skilled job but could not keep it. In May 2010, she missed a planned injection of the drug and her condition worsened, the behavioural disorders increased and she began to run away from home. She was examined by a district psychiatrist during a home visit. The doctor noted anxiety, delusions and hallucinations ('smells were suspicious'' 'voices', 'asked not to send her signals', she would speak to someone who was not there), the patient was aggressive towards her mother and expressed no awareness of her condition. She was admitted to a psychiatric hospital for one month (May to June 2010) and received risperidone therapy. After stabilization of her condition, she was discharged with a recommendation to continue taking risperidone at 2 mg per day. Subsequently, she was observed by a district psychiatrist and received risperidone at a dose of 6 mg per day and trihexyphenidyl at a dose of 4 mg per day. Due to the fact that the patient developed extrapyramidal symptoms, the dose of risperidone was reduced to 3 mg per day. The patient was also prescribed a course of diazepam injections of 10 mg intramuscularly for five days, with further transfer to the tableted form of low doses of benzodiazepines at night for a month. This treatment had a positive effect. The patient's condition remained stable for eight years and could be regarded as medically induced remission without admissions to a psychiatric hospital or a psychoneurological dispensary. However, social maladjustment persisted, and the patient could not find a job. Over time, social and domestic incompetence and emotional-volitional decline increased. In 2012, the Disability Determination Services classified the patient into disability group II for lifetime. In November 2018, she again cancelled maintenance therapy and was admitted to a psychiatric hospital with hallucinatory-delusional symptoms, where she was treated for three weeks. She was discharged with a recommendation to continue treatment with risperidone at a dose of 2 mg before bed, followed by transfer to intramuscular administration of the long-acting suspension of risperidone at a dose of 37.5 mg once every two weeks. In May 2019, she was transferred to injectable paliperidone palmitate at a dose of 75 mg once a month.

State at the moment of examination: During the year of taking injections of paliperidone palmitate at a dose of 75 mg once a month, no exacerbations were observed. The patient lives with her mother, family relations are good, she helps with the housework, is looking for a job, attends interviews and is registered with the employment centre. She regularly visits a psychoneurological dispensary, where no adverse effects of antipsychotic therapy have been registered. Her mental state can be described as a sustained medically induced remission for more than a year.

* - In the description of clinical cases, the personal data of the patient and the places of his treatment are not given.

** - In Russia, there are three disability groups that are assigned depending on performance incapability. A disability group can be assigned for a certain period of time or for the whole lifetime.

#### Discussion

Noncompliance is a common problem in patients with schizophrenia, which is the most common cause of relapse. The above clinical case demonstrates problems of compliance with the treatment regimen and associated relapses and repeated hospitalizations during disease progression. The long-acting antipsychotics reduce the frequency of drug admission and, therefore, can effectively improve compliance. Research conducted by Global Data shows that physicians often prefer long-acting injectable antipsychotics to pills, especially in patients with a history of non-compliance. In the described case, switching to a first-generation long- acting antipsychotic could not solve this problem, because of side effects that were difficult for the patient to tolerate. The transfer to a second-generation long- acting injectable antipsychotic neutralized the adverse effects and facilitated adherence to therapy. The intended effect of PP1M treatmeet is to ensure the social functioning of patients, especially work and study, interpersonal contacts, self-care and the elimination of disorganized behaviour and aggression. This clinical case demonstrates not only the possibility of ensuring compliance in a patient who is prone to violation of the therapy regimen, but also the improvement of social adjustment, improvement of functioning and quality of remission, and an effective impact on negative symptoms in a patient when transferring to paliperidone palmitate in injectable form at a dose of 75 mg once a month.

### CLINICAL CASE 2

Patient N., born in 1966 (age 54) in the Leningrad region, the second child in the family. She has no known family history of mental disorders. Her mother is a paramedic, and her father was an engineer (he died at an advanced age of cardiovascular morbidity). The patient did not have any disturbances of early development or adolescence. Menstruation started from the age of 14, with a regular cycle. She went to school at the age of seven and studied satisfactorily, with a preference for humanities. Following graduation from school, she entered a medical institute, after which she worked as a paediatric neurologist in a hospital for many years. In 2000, she got married and her son was born. In 2002 she got divorced from her husband, as they 'turned out to be incompatible'. Overall, she had four pregnancies and three abortions. She experiences the onset of menopause at the age of 54.

The patient noted a change in her mental state after childbirth, when she became depressed and indifferent, but she did not seek medical help. According to her statement, 'everything cured itself. In 2003, she began to notice sleep disorders, woke up 'as if pushed'and became irritable. She sought a psychotherapist, who prescribed amitriptyline at a dose of 25 mg before bed for two weeks. Her state improved and subsequently, she took this medication occasionally when sleep was disturbed. A year later, she began to experience the effects of 'electrical stimuli from the ambient environment', in the form of an unpleasant physical feeling, 'contraction' of the scalp 'as if the brain is moving'. The patient self- medicated using the fabomotizol, tofisopam and pyracetam. During this period, she moved to Saint Petersburg (2004). She got a job as a doctor, but worked for less than six months before quitting and subsequently changed jobs frequently for delusional reasons, as she 'felt an influence everywhere'. She had disturbed sleep, constantly experienced anxiety and depression, and 'sat at the table, looking at nothing for several hours'. She has been unemployed since 2007. She became withdrawn, uncommunicative, abandoned the household, stopped cooking and expressed delusional ideas that her mother and her son were not her own relatives. She suspected that she was under 'surveillance [and] wiretapping'. She refused the help of medical specialists and started attending church. In March 2008, she was admitted to a psychiatric hospital for three months (March to June 2008). On the day of her hospitalization, she opened a balcony window, shouting 'darling, I love you, I'm coming to you'. She could not explain her behaviour. She had incoherent monologic speech, said that she was hearing 'voices', was agitated, anxious and suspicious. She looked around, paused and strained her ears to hear something. She expressed delusional ideas, saying 'I'm a doctor of the highest category, I was put here especially, I'm in the way of someone'. Diagnosis: F20.0 Paranoid schizophrenia.

In the inpatient department, she received haloperidol at a dose of 20 mg per day with further transfer to the long-acting injectable form of haloperidol at a dose of 50 mg once every 10 days and trihexyphenidyl at a dose of 6 mg per day (due to extrapyramidal symptoms). She made no complaints, kept to herself in the ward and communicated with other patients in a formal manner. Clinical assessment revealed auditory hallucinations, delusions of persecution, influence, relationships and love charm. The patient was assigned to disability group II for life. She was discharged and referred for further observation in a local psychoneurological dispensary, where she received maintenance therapy with the long-acting injectable form of haloperidol at a dose of 100 mg intramuscularly once every 14 days. The course of the disease was shiftlike. The patient's mental state remained stable for a long time, but in 2010 she began to notice apathy, depressed mood and 'inhibition of thoughts'. She was transferred to risperidone therapy at a dose of 6 mg per day. She noted an increase in activity during the day, her mood improved and she expressed explicit awareness of her condition. She regularly visited the psychoneurological dispensary, had partial insight and socialized to an extent; she got a job as a medical statistician and coped with her work well. She was again admitted to a psychiatric hospital in July 2012 because of hallucinatory- delusional symptoms and psychomotor agitation. When examined in the ward, she was suspicious, strained her ears to hear something and confirmed that she was experiencing auditory hallucinations. She received haloperidol at a dose of 20 mg per day and was subsequently transferred to the long-acting injertable form of haloperidol at a dose of 100 mg intramuscularly once every 14 days and trihexyphenidyl at a dose of 4 mg per day. At the time of discharge, the patient's state was satisfactory, but when visiting the psychoneurological dispensary, she stated that she could not work after haloperidol injections, she experienced loginess and indifference. In December 2012, she was successively transferred to the injectable form of risperidone suspension at a dose of 37.5 mg intramuscularly once per month, then to an injectable form of paliperidone suspension at a dose of 75 mg intramuscularly once per month. The patient's mental state improved and she coped with her job well. In June 2015, after the workload at work increased, she became anxious, had difficulties with concentration and developed sleep disorders, asthenia and depressive symptoms. After 30 days of treatment in a day hospital, her condition improved, she did not experience delusions and hallucinations, and no suicidal thoughts were present. She received the injectable form of risperidone suspension at a dose of 100 mg intramuscularly once a month and amitriptyline in tablet form at a dose of up to 150 mg per day. On this therapy, the negative symptoms became more visible. The patient was transferred to PP3M at a dose of 350 mg intramuscularly.

State at the moment of examination: the patient's mental state is stable, determined as a medically induced remission. She continues to work and has learned how to use a new computer program. She does not miss doctor's visits or injections and is satisfied with her general state. Hallucinatory-delusional symptoms are not detected.

#### Discussion

The above clinical case demonstrates that the transfer from a first-generation long-acting injectable to a second-generation long-acting injectable drug contributed to improving the quality of remission and its stability. There were no relapses that required hospitalization. The progressive improvement in social functioning was noted, especially when transferring to the injectable form of paliperidone suspension at a dose of 350 mg intramuscularly once every three months.

### CLINICAL CASE 3

Patient A., born in 1958 (age 62) in Leningrad. Her mother suffered from mental health problems, was often treated in a psychiatric hospital and died at the age of 57. Her father was addicted to alcohol. The patient did not have any disturbances of early development and adolescence. Menstruation started from the age of 11, with a regular cycle. She started school at the age of seven and studied well. She finished 10 classes and tried to enter a higher education institute, but did not pass the entrance test and 'cried a lot, worried'. Later, she graduated from a technical university and worked as an engineer at a factory for a year. She got married at the age of 23 and her husband worked in a tram and trolleybus fleet. She had five pregnancies, with one childbirth and four abortions. She experienced menopause from the age of 50.

The patient noticed changes in her condition at the age of 21 (1979), when she went on a solo vacation to the seaside. According to the patient, during the vacation period she dated a man, a local resident, who went on to blackmail and harass her. After returning home, she told her relatives that she was constantly being called at the door and on the phone, saying 'it is still the same lover trying to find me'. At the same time, the patient herself could not contact this man, as 'he always called from different cities, constantly moved'. She was waiting for her fiance to return from the army. She constantly felt a sense of guilt, a 'guilty conscience that I cheated on him'. Her sleep was disturbed, she became suspicious and 'constantly looked around'. During this period, a psychosis developed acutely: she experienced visual hallucinations in the form of 'a blurry picture, like slow-motion video, I saw children playing volleyball in this video', it began to seem that 'all the people around are getting old, and my fiance is young, beckoning me to follow him'. She was afraid that she would turn 'into another being'. It seemed to her that she 'jumped through many years and lives in a different political system', and she was agitated. She was admitted to a psychiatric hospital. Upon admission, she developed hallucinatory-delusional symptoms and mental automatism, shouting 'my fiance is calling me, I must be with him', spoke to herself and showed verbal aggression. She received therapy with a solution of insulin for subcutaneous injection and thioridazine in tablet form (no information is available on dosages of these medications). Her mental state was unstable, her behaviour was childish and she continued to experience hallucinatory-delusional symptoms from time to time. She experienced tension, anxiety and ollfactory hallucinations appeared, which she described as 'it seemed that everything around me is rotting'. She began to tell that she 'split up, physically she was in the ward but her brain was in the dining room'. Trifluoperazine in the form of a 0.2% solution for intramuscular injection and small doses of haloperidol (per os) were added to treatment, after which her mental state improved. Diagnosis: F20.0 Paranoid schizophrenia.

After discharge from hospital, she received maintenance therapy with haloperidol at a dose of 1.5 mg per day, trihexyphenidyl at a dose of 4 mg per day and trifluoperazine at a dose of 10 mg per day. She worked as a cleaner, raised her son and ran a household. Until 1988, the mental state was determined as medically induced remission. Several years later, the emotional-volitional decline increased, the patient was repeatedly treated in a day hospital because of the development of paranoid symptoms and was transferred to olanzapine therapy. Negative symptoms were highllghted by clinical assessment. She stopped working and at the age of 42 (2000), she was assigned to disability group II for life. In May 2004, she was admitted to a psychiatric hospital because of a relapse, in which she stopped sleeping, began to attend church and expressed delusional ideas of sinfulness, of 'possession by the Devil'. On the day of admission to the hospital, she jumped from a third floor balcony and was hospitalized involuntarily. She was discharged after two months with a recommendation of clozapine in tablet form at a dose of 150 mg per day. She received this maintenance therapy for a year, during which time she felt well. Her mental state worsened in December 2007, with the appearance of sleep disorder and abnormal preoccupations. She was treated in a day hospital from December 2007 to June 2008, with risperidone at a dose of 6 mg per day, followed by transfer to risperidone for intramuscular injection at a dose of 37.5 mg once every two weeks. The patient was discharged in a satisfactory mental state and got a job as a cleaner. In 2012, her mental state deteriorated again with the appearance of neurosis-llke symptoms and she was prescribed clozapine therapy at a dose of 150 mg per day in a day hospital. After discharge, while taking this drug she complained of apathy, a lack of initiative and indifference. In December 2013, she was transferred to a suspension of paliperidone palmitate at a dose of 50 mg once a month, with a further increase in dose to 75 mg per month. In the following years, she was not hospitalized, her state was determined as stable medically induced remission and she has been in employment. In July 2017, she was transferred to PP3M at a dose of 263 mg intramuscularly.

State at the moment of examination: on the maintenance antipsychotic therapy, the patient notes an increase in activity, runs a household in the summer cottage, continues to work, her family relations have improved and she lives with her husband and son. Currently, the patient's state is determined as a stable medically induced remission.

#### Discussion

It can be noted that the administration of risperidone in injectable form to the patient contributed to the formation of a fairly stable medically induced remission for four years. The change of therapy (clozapine prescription) was apparently due to an increase in anxiety, which was regarded as signs of relapse, but psychosis did not develop and hospitalization was not required. Poor tolerability of clozapine, which significantly reduced the patient's quality of life, led to the decision to transfer to PP1M, leading to a decrease in antipsychotic therapy side effects which improved the quality of remission along with effective control of schizophrenia symptoms. The transfer to PP3M led to further improvement in the patient's mental state and quality of remission. This clinical case illustrates the possibility of a noticeable improvement in the social functioning of a patient with chronic schizophrenia, combined with good tolerability of second-generation long-acting injectable antipsychotics.

### CLINICAL CASE 4

Patient T.. born in 1982 (age 38) in Leningrad, the only child in the family. Her maternal grandparents abused alcohol. Her mother is hyperactive, in the past she worked as a librarian, a guide and later ran her own real estate agency. Her father, according to the patient, was 'ideal': calm and good-natured. At the same time, her father has another family and the patient was born out of wedlock. She maintains a relationship with her father. She was raised by her mother was overprotective. The patient did not have any disturbances of early development or adolescence. Menstruation started from the age of 14, with a regular cycle.

In early childhood, the patient did not have problems with social adjustment. From the age of 10 months, she attended a nursery, then a kindergarten. She started school from the age of seven, attended various groups (drawing, dancing and gymnastics), but left the groups 'because of shyness'. The patient stated that 'my mother said that I have always been a sickly child' because she was diagnosed with biliary dyskinesia, which did not bother her in any way, 'but my mother constantly stuffed me with all sorts of medications'. After school, she wanted to go to a medical university, but at the insistence of her mother, she entered a faculty of foreign languages of a pedagogical institute. She graduated from the institute 'with difficult, because I fell in love'. For a year after graduation, she worked as an English teacher at a medical school, then quit. After that, she taught English privately for several years. In 2009, she gave birth to a daughter out of wedlock and did not maintain relationship with the child's father. The patient noted that since childhood she was 'thoughtful, withdrawn, shy, afraid of everything'.

Around the age of 19 (2001), she noticed a high mood, became hyperactive, attended various entertainment events, 'strongly fell in love' with a university professor, stopped spending time studying, 'mooned over him' and 'thought that she would dance with him in a ballet all over the world'. During that period, the patient's mother began to notice that her daughter's behaviour changed; she became secretive, 'started a diaty, kept writing in it'. After the professor left the institute, she 'continued to seek his affection', called him, found out where he lived, 'began to go there, wait for him' and wrote about him in her diary: 'I must give birth to his son, and then our planet will be saved, I will give birth to the Messiah'. This state lasted for more than a year and she barely graduated from the institute. In 2006, she became aggressive and quick-tempered. She tried to find the professor. She believed that her mother wanted to cause her harm 'so she does not allow her to arrange her personal life', 'envies that I am the chosen one', noted that 'everyone is in cahoots, they want to kill me', became suspicious and stopped going outside. At that time, she lived alone, called her mother in tears, saying that 'someone is standing outside my door and wants me dead', had a feeling that 'she was dying', felt her heartbeat and was afraid that 'her breathing would stop'. She stopped eating as it seemed to her that 'the food could have been poisoned'. In May 2006, she was first admitted to the university psychiatric clinic. Upon admission, she developed hallucinatoty-delusional symptoms, psychic automatism, spoke to herself and showed verbal aggression. Diagnosis: F20.0 Paranoid schizophrenia. She was on treatment for two months, during which she received trifluoperazine at a dose of 10 mg per day and trihexyphenidyl at a dose of 4 mg per day. Her mental state stabilized, abnormal preoccupations and hallucinatory- delusional symptoms reduced, and her mood stabilized. In the ward, she kept to herself, did not communicate with anyone and did not express any awareness of her state.

After discharge from hospital, the patient took maintenance therapy for a year. She began to engage in private teaching, but after a period of work overload, her sleep was disturbed and she became irritable and anxious. The dose of trifluoperazine was increased to 20 mg per day. She began to notice tremors, 'problems with the tongue [and] jaw' and felt stiffness of muscles. In November 2007, she was transferred to risperidone therapy at a dose of 4 mg per day in the psychoneurological dispensary. She continued to experience extrapyramidal symptoms, so she stopped taking the drug on her own. She did not receive the maintenance therapy for two years. During this period, she met a man and got pregnant. According to her mother, during pregnancy, 'she started indulging in illusions again' and was sure that the child's father was actually her professor, but her behaviour was not disturbed and she was calm. She gave birth to a daughter in 2009, out of wedlock. After the birth, she did not maintain a relationship with the child's father, saying 'he just vanished, who cares'; she did not worry, she 'was up in the clouds' and took care of the child under the supervision of her mother. A few months after birth, she began to express ideas of reference towards her mother, blamed her that 'her husband left her, she was jealous of me, she was in love with him and broke our relationship', became angry, irritable and decided to find her professor, 'because this child had to be his'. She stopped taking care of the child, did not feed her and according to the patient's mother 'the child did not bother my daughter in any way'. When the patient's mother wanted to take the child into her care, the patient showed aggression, saying 'you're a witch, you want to kill my child'. She became suspicious, checked the food again because she thought that 'her mother might have poisoned her', listened to sounds coming from the street at the window, drew the curtains, closed the windows in the room and sat in silence for a long time 'so as not to give herself away'. She reported that 'they show me on the Internet, they know what shampoo I use' and turned off lights in the apartment. She began to think that she was being 'shot on camera'. In September 2009, she was admitted to a psychiatric hospital involuntarily. During the transportation, she resisted, cursed and shouted 'you are all in collusion, I did not give birth to the Messiah, you want to kill me'. Upon admission, she developed hallucinatory-delusional symptoms. In hospital, she received risperidone at a dose of 2 mg per day. During the therapy, her condition stabilized and hallucinatory-delusional symptoms were resolved. Thought disorders, negative symptoms and increasing apathy and abulia symptoms came to the forefront in clinical assessment. In the ward, she kept to herself, did not communicate with other patients and did not make plans for the future. After discharge, she was assigned to disability group II for a year. Her mental state was stable on the maintenance therapy for several years, but negative symptoms and personality changes came to the forefront in clinical assessment, and autism symptom were growing. She was engaged in raising her daughter together with her mother, and the relationship in the family was satisfactory. In 2011, she stopped taking medication, became wary and anxious, relations with her neighbours and her mother worsened, she stopped taking her child to kindergarten, was afraid to go out and expressed delusional ideas of persecution and exposure. She began to leave the house at night, returning in the morning with no explanation and stopped cleaning her room. In her diary, she addressed 'God, the angels' and 'the people who have been following her for many years'. She wrote about 'information leaks, loss of energy and devil worship'. She began to believe that 'her mother is in cahoots with the devil worshippers'. She reported that there was an invasion of her life, that someone 'is constantly filming her on camera, laughing at her', asked to 'turn off the broadcast of me' and that 'these are experiments on me'. The patient was involuntarily admitted to a psychiatric hospital. Upon admission, she developed hallucinatory-delusional symptoms, was agitated and screamed 'Freemasons, stop trying to force powerful thoughts on me'. She tried to escape, saying 'I must jump from a roof and die'. In the ward, she was tense, negatively predisposed, anxious, suspicious, psychomotor agitation persisted and periodically increased, she was impulsive, spiteful and her speech was in monologue. She revealed pronounced disorders ofthinking, expressed affective delusional ideas of reference, persecution, significant influence and gave a delusional interpretation of what was happening, considering hospitalization to be the result of 'collusion of doctors with my mother", reporting that her mother'spoils my energy, wants to replace my aura with hers' and accusing her of 'practising black magic'. At first, she received therapy with haloperidol at a dose of up to 10 mg per day, but noted pronounced extrapyramidal symptoms and was transferred to therapy with quetiapine at a dose of 600 mg per day. Her state showed some improvement, but a week after starting quetiapine, she again began to express delusional ideas. She was transferred to risperidone at a dose of up to 9 mg per day and trihexyphenidyl at a dose of 4 mg per day. On this therapy, her state improved, her behaviour was ordered, delusional symptoms stopped and extrapyramidal symptoms were reduced.

After discharge, the patient was observed by a district psychiatrist and received maintenance therapy. Her condition remained stable for a long time, determined as a stable medically induced remission for three years without hospitalization. She got a low-skilled job (as a cleaner) and helped her mother to raise her daughter. However, she experienced personality changes along with increases in social and domestic incompetence and emotional-volitional decline. In March 2014, she again cancelled the maintenance therapy herself and her mental state worsened. She was admitted to a psychiatric hospital with hallucinatory-delusional symptoms. After treatment, she was discharged with a recommendation for risperidone at a dose of 9 mg per day and trihexyphenidyl at a dose of 4 mg per day, before being transferred in June 2014 to long-acting risperidone at a dose of 37.5 mg once every two weeks. This dose was later increased to 50 mg once every two weeks. In April 2015, the patient was transferred to PP1M at a dose of 100 mg intramuscularly. She regularly visited the psychoneurological dispensary, did not miss injections, was not hospitalized, became more active, helped her mother with the household, began to give private English lessons and occasionally worked as a cleaner. In March 2020, she was transferred to PP3M at a dose of 263 mg intramuscularly.

State at the moment of examination: on the maintenance antipsychotic therapy the patient's condition is stable, she continues to work, has plans for the future, family relations have improved and she has become more caring for her daughter. Her condition is determined as a stable medically induced remission.

#### Discussion

Clinical case 4 illustrates the stabilization of the patient's state on PP1M with high medication tolerability. The following transfer to PP3M—Trevicta—brings benefits of treatment adherence, which helps to maintain a stable remission, prevent rehospitalization and improve social functioning of the patient.

The above clinical cases confirm previous data in the literature that treatment with PP1M and PP3M enables a more stable mental condition. There is evidence that since patients are in a more stable state, the burden of disease with treatment of PP1M and PP3M is lower than from usual treatment, as it reduces the burden on psychiatric services and the hospital sector. When treated with these medications, the burden associated with a person's disease is outweighed by the benefits to their health [15] [16].

According to the literature, in real clinical practice, patients with schizophrenia who are transferred from PP1M to PP3M show a decrease in the use of health resources and an increase in treatment adherence within months of starting PP3M, as well as adherence to PP3M therapy [8]. All the clinical case studies we have presented are consistent with previous research findings and illustrate a steady reduction in negative symptoms, reflected by improvement in the functioning and psychosocial adjustment of patients and effective relapse prevention.

## CONCLUSION

Second-generation long-acting injectable antipsychotics can provide control of psychopathology to improve social recovery and quality of life of patients and their families, reduce the burden of disease on both the health system and families or caregivers. The experience of using paliperidone palmitate indicates that this drug allows patients to maintain a stable state for a long time and reduce the severity of negative disorders. When using paliperidone palmitate, there are no adverse events in the form of excessive sedation and loginess, or extrapyramidal symptoms. This contributes to adherence to therapy and destigmatization of patients with schizophrenia. The clinical cases presented here demonstrated an improvement in the quality of remission of schizophrenia due to high anti-relapse effectiveness, efficacy against negative symptoms, safety and high tolerability of PP1M and PP3M regardless of patient age. We may conclude that PP3M is no less effective than PP1M.

## Authors contributions

Nataliia N. Petrova: concept, theoretical analysis, review of recent publications, manuscript draft; Valeriia S. Serazetdinova: clinical inputs, selection of illustrative case reports.

## Conflicts of Interest

The authors have no conflict of interest to declare.

## Funding

The research has no sponsors.
